# *Escherichia coli* Sequence Type 131 *H*30 Is the Main Driver of Emerging Extended-Spectrum-β-Lactamase-Producing *E*. *coli* at a Tertiary Care Center

**DOI:** 10.1128/mSphere.00314-16

**Published:** 2016-11-23

**Authors:** James R. Johnson, Brian Johnston, Paul Thuras, Bryn Launer, Evgeni V. Sokurenko, Loren G. Miller

**Affiliations:** a VA Medical Center, Minneapolis, Minnesota, USA; b University of Minnesota, Minneapolis, Minnesota, USA; c Los Angeles BioMedical Research Center at Harbor–Harbor-UCLA Medical Center, Torrance, California, USA; d University of Washington, Seattle, Washington, USA; e University of California, Los Angeles, California, USA; JMI Laboratories

**Keywords:** *Escherichia coli* infections, ST131, ST131-*H*30, antimicrobial resistance, clonality, extended-spectrum β-lactamase, fluoroquinolone resistance, molecular epidemiology, phylogenetic analysis

## Abstract

The *H*30 strain of *Escherichia coli* sequence type 131 (ST131-*H*30) is a recently emerged, globally disseminated lineage associated with fluoroquinolone resistance and, via its *H*30Rx subclone, the CTX-M-15 extended-spectrum beta-lactamase (ESBL). Here, we studied the clonal background and resistance characteristics of 109 consecutive recent *E. coli* clinical isolates (2015) and 41 historical ESBL-producing *E. coli* blood isolates (2004 to 2011) from a public tertiary care center in California with a rising prevalence of ESBL-producing *E. coli* isolates. Among the 2015 isolates, ST131, which was represented mainly by ST131-*H*30, was the most common clonal lineage (23% overall). ST131-*H*30 accounted for 47% (8/17) of ESBL-producing, 47% (14/30) of fluoroquinolone-resistant, and 33% (11/33) of multidrug-resistant isolates. ST131-*H*30 also accounted for 53% (8/14) of dually fluoroquinolone-resistant, ESBL-producing isolates, with the remaining 47% comprised of diverse clonal groups that contributed a single isolate each. ST131-*H*30Rx, with CTX-M-15, was the major ESBL producer (6/8) among ST131-*H*30 isolates. ST131-*H*30 and *H*30Rx also dominated (46% and 37%, respectively) among the historical ESBL-producing isolates (2004 to 2011), without significant temporal shifts in relative prevalence. Thus, this medical center’s recently emerging ESBL-producing *E. coli* strains, although multiclonal, are dominated by ST131-*H*30 and *H*30Rx, which are the only clonally expanded fluoroquinolone-resistant, ESBL-producing lineages. Measures to rapidly and effectively detect, treat, and control these highly successful lineages are needed.

**IMPORTANCE** The ever-rising prevalence of resistance to first-line antibiotics among clinical *Escherichia coli* isolates leads to worse clinical outcomes and higher health care costs, thereby creating a need to discover its basis so that effective interventions can be developed. We found that the *H*30 subset within *E. coli* sequence type 131 (ST131-*H*30) is currently, and has been since at least 2004, the main *E. coli* lineage contributing to key resistance phenotypes—including extended-spectrum-beta-lactamase (ESBL) production, fluoroquinolone resistance, multidrug resistance, and dual ESBL production-plus-fluoroquinolone resistance—at a United States tertiary care center with a rising prevalence of ESBL-producing *E. coli* isolates. This identifies ST131-*H*30 as a target for diagnostic tests and preventive measures designed to curb the emergence of multidrug-resistant *E. coli* isolates and/or to blunt its clinical impact.

## Abstract (precis)

The ever-rising prevalence of resistance to first-line antibiotics among clinical *Escherichia coli* isolates leads to worse clinical outcomes and higher health care costs, thereby creating a need to discover its basis so that effective interventions can be developed. We found that the *H*30 subset within *E. coli* sequence type 131 (ST131-*H*30) is currently, and has been since at least 2004, the main *E. coli* lineage contributing to key resistance phenotypes—including extended-spectrum-beta-lactamase (ESBL) production, fluoroquinolone resistance, multidrug resistance, and dual ESBL production-plus-fluoroquinolone resistance—at a United States tertiary care center with a rising prevalence of ESBL-producing *E. coli* isolates. This identifies ST131-*H*30 as a target for diagnostic tests and preventive measures designed to curb the emergence of multidrug-resistant *E. coli* isolates and/or to blunt its clinical impact.

## INTRODUCTION

*Escherichia coli* is a major extraintestinal pathogen and the most common cause of urinary tract infections (UTIs) (1). It poses increasing clinical challenges due to emerging antimicrobial resistance, most notably to fluoroquinolones and extended-spectrum cephalosporins (ESCs), with the latter mediated mainly by extended-spectrum beta-lactamases (ESBLs), such as CTX-M-15 (2, 3). The dissemination of specific drug-resistant clonal groups accounts for much of this problem (4, 5). The fluoroquinolone-resistant *H*30 subclone of *E. coli* sequence type 131 (ST131-*H*30), with its nested ESBL (CTX-M-15)-associated *H*30Rx subset, is the most dramatically expanded antimicrobial-resistant *E. coli* lineage (6–8).

In 2014, Harbor-UCLA Medical Center (Los Angeles, CA) noted a steadily rising prevalence of ESBL production among *E. coli* clinical isolates in its antibiograms over the six preceding years, from 8.7% (297/3,405) (in 2008) to 20.8% (829/3,989) (in 2013), with most ESBL-producing strains being multidrug resistant (MDR) and, specifically, having a 90% prevalence of fluoroquinolone resistance (L. Miller, unpublished data). Of note, these antibiograms did not include duplicate isolates of the same organism with the same antimicrobial susceptibility profile from the same patient within 2 weeks. Here, we sought insights into this ESBL emergence; i.e., whether it was most likely due to extensive horizontal transfer of ESBL-encoding determinants or the expansion of a single or multiple ESBL-producing lineages and, if so, which. We also sought to understand the clonal background of resistance generally.

## RESULTS

### Source and resistance.

From 2015, the 109 consecutive unique *E. coli* isolates were from urine (82%), blood (8%), and miscellaneous sources (10%); 31% were from inpatients (Table 1). Overall, 16% produced ESBLs, and 17% were PCR positive for *bla*_CTX-M_, 13% for *bla*_CTX-M_ group 1/*bla*_CTX-M-15_, and 3% for *bla*_CTX-M_ group 9. Two isolates were ESBL phenotype negative despite containing an ESBL-encoding gene. Resistance prevalence ranged from 7% (nitrofurantoin and piperacillin-tazobactam) to 69% (ampicillin) (Table 1), and resistance scores from 0 to 5 (median score of 2). Thirty percent of isolates were MDR. The ESBL-producing isolates were significantly associated with resistance to most individual agents and MDR status (Table 2) and had higher resistance scores (median score of 4, versus 1 for non-ESBL-producing isolates; *P* < 0.001). Specifically, 88% of ESBL-producing strains were also resistant to fluoroquinolones.

**TABLE 1:** Phylogenetic distribution of source and resistance traits among 109 *Escherichia coli* clinical isolates

Source or resistance trait	No. (%) of isolates with trait							*P* value^a^
	All isolates (*n* = 109)	Group A (*n* = 7)	Group B1 (*n* = 6)	Group B2 (*n* = 62)	Group C (*n* = 1)	Group D (*n* = 28)	Group F (*n* = 5)	
Source								
Urine	89 (82)	5 (71)	5 (83)	51 (82)	1 (100)	25 (85)	2 (40)	**0.025**^b^
Blood	9 (8)	2 (25)	0 (0)	5 (81)	0 (0)	2 (7)	0 (0)	NA^c^
Other	11 (10)	0 (0)	1 (17)	6 (10)	0 (0)	1 (4)	3 (60)	NA
Inpatient isolate	34 (31)	2 (29)	2 (33)	21 (34)	0 (0)	6 (21)	3 (60)	0.57
ESBL phenotype^d^	17 (16)	1 (14)	1 (17)	12 (19)	0 (0)	1 (4)	2 (40)	0.29
*bla*_CTX-M_ universal	19 (17)	2 (29)	1 (17)	12 (19)	0 (0)	2 (7)	2 (40)	0.44
*bla*_CTX-M-15_ or group 1^e^	14 (13)	2 (29)	1 (17)	9 (15)	0 (0)	0 (0)	2 (40)	**0.096**
*bla*_CTX-M_ group 9	3 (3)	0 (0)	0 (0)	2 (3)	0 (0)	1 (4)	0 (0)	0.99
Ampicillin	75 (69)	4 (57)	5 (83)	41 (66)	0 (0)	22 (79)	3 (60)	0.43
Ampicillin-sulbactam	64 (59)	3 (43)	4 (67)	38 (61)	0 (0)	17 (61)	64 (59)	0.66
Cefazolin	26 (24)	1 (14)	2 (33)	17 (27)	0 (0)	4 (14)	2 (40)	0.62
Ceftazidime, ceftriaxone^f^	19 (17)	1 (14)	2 (33)	13 (21)	0 (0)	1 (4)	2 (40)	0.20
Ciprofloxacin, levofloxacin^f^	30 (28)	1 (14)	1 (17)	25 (40)	0 (0)	1 (4)	2 (40)	**0.01**
Gentamicin, tobramycin^f^	13 (12)	1 (14)	1 (17)	11 (18)	0 (0)	0 (0)	0 (0)	0.24
Nitrofurantoin	6 (7)	0 (0)	3 (60)	1 (2)	0 (0)	2 (8)	0 (0)	**<0.001**
Piperacillin-tazobactam	8 (7)	0 (0)	0 (0)	7 (11)	0 (0)	1 (4)	0 (0)	0.62
Trimethoprim-sulfamethoxazole	14 (13)	1 (14)	1 (17)	11 (18)	0 (0)	0 (0)	1 (20)	0.31

a *P* values (by chi-square test, for the overall 6-group comparisons) are shown in boldface if *P* < 0.10.

b Source is stratified into three categories. *P* values are for the overall (6-group × 3-source) comparison.

c NA, not applicable (*P* value for overall 3-source comparison is shown above).

d ESBL, extended-spectrum beta-lactamase.

e The *bla*_CTX-M-15_ and *bla*_CTX-M_ group 1 primers gave identical results.

f Highly similar results were obtained for ceftazidime versus ceftriaxone, ciprofloxacin versus levofloxacin, and gentamicin versus tobramycin.

**TABLE 2:** Characteristics of 109 *Escherichia coli* clinical isolates in relation to extended-spectrum-beta-lactamase production

Source or resistance trait	No. (%) of isolates with trait			*P* value for ESBL producers versus others^a^
	All isolates (*n* = 109)	Non-ESBL-producing isolates (*n* = 92)	ESBL-producing isolates (*n* = 17)	
Source				
Urine	89 (82)	78 (85)	11 (68)	**0.10**^b^
Blood	9 (8)	7 (8)	2 (12)	NA^c^
Other	11 (10)	7 (8)	4 (24)	NA
Inpatient isolate	34 (31)	26 (28)	8 (47)	0.16
Ampicillin	75 (69)	58 (63)	17 (100)	**0.001**
Ampicillin-sulbactam	64 (59)	50 (54)	14 (82)	**0.04**
Cefazolin	26 (24)	9 (10)	17 (100)	**<0.001**
Ceftazidime, ceftriaxone^d^	19 (17)	3 (3)	16 (94)	**<0.001**
Ciprofloxacin, levofloxacin^d^	30 (28)	15 (16)	15 (88)	**<0.001**
Gentamicin, tobramycin^d^	13 (12)	4 (4)	9 (53)	**<0.001**
Nitrofurantoin	6 (7)	6 (8)	0 (0)	1.0
Piperacillin-tazobactam	8 (7)	7 (8)	1 (6)	1.0
Trimethoprim-sulfamethoxazole	14 (13)	37 (40)	11 (65)	**0.07**
Multidrug resistance (≥3 classes)	33 (30)	16 (17)	17 (100)	**<0.001**

a *P* values (by Fisher exact test) are shown in boldface if *P* ≤ 0.10.

b Source is stratified into three categories. *P* value is for the overall 3-source comparison.

c NA, not applicable (*P* value for overall 3-source comparison is shown above).

d Highly similar results were obtained with ceftazidime versus ceftriaxone, ciprofloxacin versus levofloxacin, and gentamicin versus tobramycin.

### Phylogenetic and clonal background.

The isolates were predominantly from groups B2 (57%) and D (27%); groups A, B1, C, and F were each represented by ≤7% of isolates (Table 1). ST131 (23%), represented mainly by ST131-*H*30 and its subclone *H*30Rx (14% and 9%, respectively), was by far the most prevalent clonal group, followed by sequence type complex 69 (STc69) (17%), STc95 and STc14 (11% each), STc73 (8%), STc405 (6%), and STc141 (3%), with STc12, STc127, STc372, and STc648 contributing only 1 or 2 isolates each.

### Clonal distribution of source and resistance.

The major phylogenetic groups accounted minimally for variation in source and antimicrobial resistance (Table 1). In contrast, nearly all clonal groups or subclones were significantly or borderline significantly associated with at least one source/resistance variable (Table 3). ST131-*H*30, with its *H*30Rx subclone, exhibited among the highest values for the prevalence of resistance and accounted for the greatest share of resistant isolates, including 47% of ESBL producers and of those with *bla*_CTX-M_ group 1/*bla*_CTX-M-15_ or resistance to ESCs, fluoroquinolones, gentamicin, piperacillin-tazobactam, or tobramycin. In contrast, the non-*H*30 ST131 isolates generally exhibited low values for the prevalence of resistance that, with few exceptions, did not differ from those of the rest of the population. Within ST131-*H*30, the prevalence of ESBL production and *bla*_CTX-M-15_ was greatest for *H*30Rx. Similarly, STc141, despite its small number of isolates (*n* = 3), exhibited multiple significant or borderline-significant associations with source and resistance, and STc405 was borderline significantly associated with trimethoprim-sulfamethoxazole (TMP-SMX) resistance. In contrast, for one or more agents, STc95, STc73, and STc69 were associated negatively with resistance.

**TABLE 3:** Clonal group distribution of source and resistance traits among 109 *Escherichia coli* clinical isolates

Source or resistance trait	No. (%) of isolates with trait^a^										
	Total isolates (*n* = 109)	Group B2								Group D	
		ST131				STc95 (*n* = 11)	STc73 (*n* = 8)	STc14 (*n* = 11)	STc141 (*n* = 3)	STc69 (*n* = 17)	STc405 (*n* = 6)
		All (*n* = 23)	Non-*H*30 (*n* = 9)	*H*30 (*n* = 14)	*H*30Rx (*n* = 9)						
Source^b^											
Urine	89 (82)	19 (83)	6 (67)	13 (93)	8 (89)	10 (91)	7 (88)	10 (99)	0 (0)***	15 (88)	6 (100)
Blood	9 (8)	2 (9)	1 (11)	1 (70)	1 (11)	1 (9)	0 (0)	1 (9)	1 (33)***	2 (12)	0 (0)
Other	11 (10)	2 (9)	2 (22)	0 (0)	0 (0)	0 (0)	1 (13)	0 (0)	2 (67)***	0 (0)	0 (0)
Inpatient	34 (31)	8 (35)	3 (33)	5 (36)	3 (33)	4 (36)	1 (13)	4 (36)	3 (100)*	3 (17)	0 (0)
ESBL phenotype^c^	17 (16)	9 (39)**	1 (11)	8 (57)***	6 (67)***	0 (0)	0 (0)	1 (9)	**2 (67)**†	**0 (0)**†	1 (17)
*bla*_CTX-M_ universal	19 (17)	9 (39)**	1 (11)	8 (57)***	6 (67)***	0 (0)	0 (0)	1 (9)	**2 (67)**†	0 (0)*	1 (17)
*bla*_CTX-M-15 _or group 1^d^	14 (13)	7 (30)**	1 (11)	6 (43)**	6 (67)***	0 (0)	0 (0)	0 (0)	2 (67)*	0 (0)	0 (0)
*bla*_CTX-M_ group 9	3 (3)	1 (4)	0 (0)	1 (7)	0 (0)	0 (0)	0 (0)	0 (0)	0 (0)	0 (0)	1 (17)
Ampicillin	75 (69)	20 (87)*	8 (89)	12 (86)	8 (89)	**5 (46)**†	6 (75)	8 (73)	2 (67)	13 (77)	6 (100)
Ampicillin-sulbactam	64 (59)	18 (78)*	**8 (89)**†	10 (71)	7 (78)	5 (46)	6 (75)	7 (64)	2 (67)	9 (53)	5 (83)
Cefazolin	26 (24)	12 (52)***	3 (33)	9 (64)***	7 (78)***	**0 (0)**†	0 (0)	3 (27)	**2 (67)**†	2 (12)	1 (17)
Ceftazidime, ceftriaxone^e^	19 (17)	10 (44)***	2 (22)	8 (57)***	6 (63)***	0 (0)	0 (0)	1 (9)	**2 (67)**†	0 (0)*	0 (0)
Ciprofloxacin, levofloxacin^e^	30 (28)	15 (65)***	1 (11)	14 (100)***	9 (100)***	0 (0)*	0 (0)	10 (91)***	0 (0)	0 (0)**	1 (17)
Gentamicin, tobramycin^e^	13 (12)	7 (30)**	1 (11)	6 (43)**	5 (56)***	0 (0)	0 (0)	1 (9)	2 (67)*	0 (0)	0 (0)
Nitrofurantoin	6 (7)	0 (0)	0 (0)	0 (0)	0 (0)	0 (0)	0 (0)	1 (9)	0 (0)	1 (6)	0 (0)
Piperacillin-tazobactam	8 (7)	5 (22)**	3 (33)*	2 (14)	2 (22)	0 (0)	0 (0)	2 (18)	2 (67)*	1 (6)	0 (0)
Trimethoprim-sulfamethoxazole	14 (13)	12 (52)	5 (56)	7 (50)	5 (56)	0 (0)	0 (0)**	6 (55)	2 (67)	9 (53)	**5 (83)**†

a Comparisons (of indicated group versus all other isolates) that yielded *P* values of <0.10 (by Fisher exact test) are shown in boldface. *P* values are denoted as follows: †, *P* < 0.10; *, *P* < 0.05;**, *P* ≤ 0.01;***, *P* ≤ 0.001.

b Source is stratified into three categories. *P* values are for the overall 3-source comparison.

c ESBL, extended-spectrum beta-lactamase.

d The *bla*_CTX-M-15_ and *bla*_CTX-M_ group 1 primers gave identical results.

e Highly similar results were obtained for ceftazidime versus ceftriaxone, ciprofloxacin versus levofloxacin, and gentamicin versus tobramycin.

Resistance scores and MDR status were distributed accordingly (Table 4). Whereas no major phylogenetic group was associated significantly with either variable, ST131-*H*30 and its *H*30Rx subclone exhibited significantly higher resistance scores and MDR prevalence values than did other isolates. Notably, ST131-*H*30, with its 100% prevalence of fluoroquinolone resistance, accounted for most isolates (53%) that were dually ESBL producing and fluoroquinolone resistant. In contrast, the diverse other clonal groups that accounted for the remaining 47% of dually resistant isolates each contributed only a single isolate (not shown). Among these other clonal groups was STc14 (*n* = 11), which, despite resembling ST131-*H*30 fairly closely for the prevalence of MDR status (55%) and fluoroquinolone resistance (91%), had only one ESBL-producing representative. As for other notable features of different clonal groups, STc95 and STc73 isolates each exhibited significantly lower resistance scores and a numerically lower MDR prevalence than did other isolates.

**TABLE 4:** Phylogenetic distribution of coresistance among 109 *Escherichia coli* clinical isolates

Group or subgroup (no. of isolates in group)	Mean resistance score		*P* value^c^	No. (%) of MDR isolates^a^		*P* value^c^	No. (%) of ESBL+FQ-R isolates^b^		*P* value^c^
	Others	Group		Others	Group		Others	Group	
Group A (7)	1.9	1.4	0.47	32 (31)	1 (14)	0.67	14 (14)	1 (14)	1.00
Group B1 (6)	1.8	2.5	0.26	30 (29)	3 (50)	0.36	14 (14)	1 (17)	1.00
Group B2 (62)	1.7	1.9	0.38	11 (24)	22 (36)	0.21	5 (11)	10 (16)	0.58
Group D (28)	1.9	1.6	0.30	28 (35)	5 (18)	0.15	14 (18)	1 (4)	0.11
Group F (5)	1.8	2.0	0.79	31 (30)	2 (40)	0.64	13 (13)	2 (40)	0.14
ST131 (23)	1.5	2.9	**<0.001**	20 (23)	13 (56)	**0.004**	6 (7)	9 (39)	**0.003**
Non-*H*30 (9)				31 (31)	2 (22)	0.72	14 (14)	1 (11)	1.00
*H*30 (14)	1.6	3.4	**<0.001**	22 (23)	11 (79)	**<0.001**	7 (7)	8 (57)	**<0.001**
*H*30Rx (9)	1.7	3.8	**<0.001**	25 (25)	8 (89)	**<0.001**	9 (9)	6 (67)	**<0.001**
STc95 (11)	1.9	1.0	**0.05**	32 (33)	1 (9)	0.17	15 (15)	0 (0)	0.36
STc73 (8)	1.9	0.8	**0.04**	33 (33)	0 (0)	**0.10**	15 (15)	0 (0)	1.00
STc14 (11)	1.7	2.6	**0.06**	27 (28)	6 (55)	**0.09**	14 (14)	1 (9)	1.00
STc141 (3)	1.7	2.7	0.33	31 (29)	2 (67)	0.22	15 (15)	0 (0)	1.00
STc69 (17)	1.9	1.5	0.29	30 (22)	3 (18)	0.26	15 (16)	0 (0)	0.12
STc405 (6)	1.8	2.2	0.57	32 (31)	1 (17)	0.67	14 (14)	1 (17)	1.00

a MDR, multidrug-resistant (i.e., resistant to ≥ 3 antimicrobial classes, counting penicillins and cephalosporins separately).

b ESBL+FQ-R, extended-spectrum-beta-lactamase producing and fluoroquinolone resistant.

c *P* values (by two-tailed *t* test or Fisher exact test) for comparisons of group members versus all other isolates are shown in boldface where *P* ≤ 0.10.

### Historical ESBL-producing isolates.

The temporal stability of clonal relationships was assessed among the institution’s archived ESBL-producing bloodstream isolates (2004 to 2011) (Table 5). Over this 7-year period, the prevalence of extended-spectrum-cephalosporin resistance among the institution’s *E. coli* clinical isolates rose steadily, from 3% (among 3,106 total isolates in 2004) to 13% (among 3,463 total isolates in 2011), according to the institution’s cumulative antibiogram. Clonal analysis of prospectively archived ESBL-producing bloodstream isolates from these years showed that, notwithstanding minor fluctuations, the overall clonal distribution remained fairly stable, with ST131-*H*30 and *H*30Rx predominating (46% and 37%, respectively). No other single clonal group, including non-*H*30 ST131 isolates, was nearly so prominent among the historical ESBL isolates as ST131-*H*30.

**TABLE 5:** Clonal distribution and *bla*_CTX-M_ variants by year among 41 historic and 17 recent extended-spectrum-beta-lactamase-producing *Escherichia coli* clinical isolates^a^

Trait	No. (%) of isolates with trait									
	Historic isolates^*b,c*^									2015 isolates (*n* = 17)^c^
	Total (*n* = 41)	2004 (*n* = 3)	2005 (*n* = 3)	2006 (*n* = 4)	2007 (*n* = 3)	2008 (*n* = 2)	2009 (*n* = 1)	2010 (*n* = 11)	2011 (*n* = 14)	
Group A	10 (24)	2 (67)	0	1 (25)	0	0	1 (100)	3 (27)	3 (21)	1 (6)
Group B2	22 (54)	1 (33)	3 (100)	3 (75)	3 (100)	1 (50)	0	5 (45)	6 (43)	12 (71)
ST131/O25b	21 (51)	1 (33)	3 (100)	2 (50)	3 (100)	1 (50)	0	5 (45)	6 (43)	9 (53)
Non-*H*30	2 (5)	0	0	0	1 (25)	0	0	0	1 (7)	1 (6)
* H*30	19 (46)	1 (33)	3 (100)	2 (50)	2 (67)	1 (50)	0	5 (45)	5 (36)	8 (47)
* H*30Rx	15 (37)	0	3 (100)	1 (25)	2 (67)	1 (50)	0	5 (45)	3 (21)	7 (41)
Group C	1 (2.4)	0	0	0	0	0	0	0	1 (7)	0
Group D/ST405	5 (12)	0	0	0	0	1 (50)	0	3 (27)	1 (7)	1 (6)
Group F/ST648	3 (7)	0	0	0	0	0	0	0	3 (21)	2 (12)
CTX-M-15	25 (61)	0	3 (100)	2 (50)	3 (100)	1 (50)	1 (100)	9 (82)	6 (43)	13 (76)
CTX-M-9 group	8 (20)	0	0	1 (25)	0	1 (50)	0	2 (18)	4 (29)	3 (18)

a For 2004 to 2011, all isolates were from blood. For 2015, two isolates were from blood and 15 from urine.

b For 2004 to 2009 (combined) versus 2010 to 2011 (combined), *P* > 0.10 for all comparisons.

c For 2004 to 2011 (combined) versus 2015, *P* > 0.10 for all comparisons.

## DISCUSSION

The findings of this study, which analyzed 109 recent and 41 historical clinical *E. coli* isolates from a United States tertiary care center with a rising prevalence of ESBL-producing *E. coli* isolates, confirm the strong linkage of specific *E. coli* clonal groups with antimicrobial resistance (4, 5, 9). Here, lineage-specific associations were much stronger and more numerous than associations involving major *E. coli* phylogenetic groups, which illustrates the value of “drilling down” to biologically relevant subspecific taxa, in contrast to the standard clinical laboratory practice of identification only to the species level (5, 9). Importantly, the observed lineage-specific associations included both exceptionally high and low resistance prevalences and scores (5, 9). Such lineage-specific susceptibility data, if combined with rapid determination of clonal group (9), could be used clinically for selecting optimal patient-specific antimicrobial therapy, conceivably even at the point of care.

The high prevalence of ST131-*H*30 and its *H*30Rx subclone, both overall and especially among resistant isolates, has potentially important practical implications. First, the prominence of *H*30Rx among the present and historic ESBL-producing isolates (35% to 37%) suggests that the institution’s recent surge in ESBL-producing *E. coli*, while polyclonal, largely represents the further expansion of this pandemic MDR lineage, rather than new emergence of other ESBL-producing lineages or widespread horizontal gene transfer. This implies that focused attention to this single strain, including through rapid detection (9), transmission blockade, and/or reservoir elimination, could help in reducing ESBL infections. Notably, our archival findings from 2004 to 2011 contrast with similar data from Calgary from 2000 to 2010 that show a progressively increasing ST131 fraction among ESBL-producing *E. coli* bloodstream isolates (10); this suggests that the dynamics of clonal emergence may vary geographically.

Second, apart from ESBL production, ST131-*H*30 accounted for approximately half of the study’s 2015 fluoroquinolone-resistant isolates, similar to results from other centers (5, 8). This suggests that efforts to reduce fluoroquinolone resistance-related morbidity and costs should include attention to this strain. The clinical importance of the combined ESBL production and fluoroquinolone resistance phenotype, which here was strongly associated with ST131-*H*30 and *H*30Rx, has been noted recently for patients with acute pyelonephritis (3).

Additionally, STc14 (O75 associated), also from group B2, accounted for two-thirds of the present non-ST131-*H*30 fluoroquinolone-resistant isolates. STc14 has been associated previously with antimicrobial resistance, including to ampicillin (11) and fluoroquinolones (12). Interestingly, here STc14 did not contribute appreciably to the clonal expansion of ESBL-producing strains, indicating that while ESBL production is associated closely with fluoroquinolone resistance, the opposite is not necessarily true. The discovery of how and why ST131-*H*30 and STc14 have diverged so markedly with respect to their resistance profiles, both from other group B2 lineages and from each other, could prove valuable.

Also notable were three group D-associated lineages—STc69, STc405, and STc31. STc69, initially called “clonal group A,” appeared in the 1990s as a widespread cause of TMP-SMX-resistant UTIs (13, 14). Here, STc69, although prominent (as the next-most-prevalent clonal group after ST131 and slightly more prevalent than ST131-*H*30), was associated with antimicrobial susceptibility. STc405, noted recently as an emerging MDR clone (15), appeared comparatively benign, except for a borderline high prevalence of TMP-SMX resistance. In contrast, STc31 (also known as the O15:K52:H1 clonal group), the first MDR *E. coli* lineage known to cause a community-wide outbreak (16, 17), was absent. This aligns with STc31’s usually minor contribution to recent *E. coli* study populations, albeit sometimes in association with fluoroquinolone resistance (4, 18).

The study has limitations. First, ESBL-encoding genes were detected by PCR but not sequenced, which leaves uncertainty as to the specific *bla* variant(s) present in several isolates; however, such data would be unlikely to change the study’s main conclusions. Second, epidemiological data were unavailable, which precluded analyses of host characteristics, clinical manifestations, and outcomes in relation to clonal background (19). In that regard, it is possible that the emergence of ESBL-producing *E. coli* at this institution was due more to expansion of the at-risk host population (e.g., elderly, compromised, and antibiotic-exposed patients) than to special bacterial strains or traits (19). Third, the historical isolates were all blood isolates, possibly biasing the comparison of historical versus recent isolates; however, most previous studies have not found clonal distribution to vary significantly in relation to specimen type (e.g., see reference 8). Fourth, the use of multiple comparisons risked finding spurious associations by chance alone, whereas the small group size for some comparisons risked missing true associations due to low statistical power.

The study also has strengths. These include the recent origin of the 2015 isolates, the inclusion of a historical comparison group obtained from 2004 through 2011, the detailed clonal typing, and the comparisons of clonal background with clinically relevant resistance phenotypes and genotypes.

In summary, among 109 recent and 41 archival clinical *E. coli* isolates from a public tertiary care center in Los Angeles, we identified strong clonal associations with antimicrobial resistance, especially for ST131-*H*30 and its ESBL-associated *H*30Rx subclone. The predominance of *H*30Rx within a polyclonal ESBL expansion likely underlies the recent surge in ESBL-producing *E. coli* isolates at this center. These clonal associations support the use of rapid diagnostics for improved prescribing and focused infection prevention efforts.

## MATERIALS AND METHODS

### Isolates and susceptibility testing.

Harbor-UCLA Medical Center is a 400-bed tertiary care public hospital; its laboratory processes specimens from patients in the hospital and from the emergency department and dozens of affiliated general and specialty clinics in the center and surrounding community. For this study, the Clinical Microbiology Laboratory provided 109 consecutive single-patient *E. coli* isolates from 2015, accompanied by data regarding antimicrobial susceptibility (obtained using Clinical Laboratory Standards Institute interpretive criteria and the Vitek II instrument [bioMérieux, Durham, NC]), specimen type, and inpatient/outpatient source. Additionally, the Infection Control Department provided all 41 available archived ESBL-producing *E. coli* bloodstream isolates from 2004 to 2011, accompanied by the corresponding annual antibiograms. These archival isolates were drawn from the Infection Control Department’s prospectively assembled collection of all sterile site isolates from the Clinical Microbiology Laboratory, which were saved regardless of species and susceptibility profile.

ESBL production was inferred from the enhancement of ESC susceptibility by clavulanate (20). Intermediate results were considered resistant. The resistance score was the number of drug classes to which resistance was detected, counting penicillins and cephalosporins separately. Isolates with resistance scores of ≥3 were considered multidrug resistant (MDR) (21).

### Molecular typing and statistical analysis.

Using duplicate boiled lysates, established and validated PCR-based assays were used to detect *bla*_CTX-M_ (universal, group 1, group 9, and CTX-M-15) (22), the *E. coli* phylogenetic group (23), 13 clonal groups (4, 24–26), and the *H*30 and *H*30Rx ST131 subclones (8, 27). Notably, in contrast to other ST131 detection assays, the assay used here is 100% sensitive and specific for this lineage (4, 28). Comparisons were tested using the chi-square or Fisher’s exact test for proportions and two-sided *t* tests for scores.
